# Non-Canonical Wnt16 and microRNA-145 Mediate the Response of Human Bone Marrow Stromal Cells to Additively Manufactured Porous 3-Dimensional Biomimetic Titanium–Aluminum–Vanadium Constructs

**DOI:** 10.3390/cells14030211

**Published:** 2025-02-01

**Authors:** David. J. Cohen, Michael B. Berger, Jingyao Deng, Thomas W. Jacobs, Barbara D. Boyan, Zvi Schwartz

**Affiliations:** 1 Department of Biomedical Engineering, College of Engineering, Virginia Commonwealth University, Richmond, VA 23284, USA; djcohen@vcu.edu (D.J.C.); bergerbme@gmail.com (M.B.B.); jingyaodeng@gmail.com (J.D.); twjacobs@vcu.edu (T.W.J.); bboyan@vcu.edu (B.D.B.); 2 Wallace H. Coulter Department of Biomedical Engineering, Georgia Institute of Technology, Atlanta, GA 30332, USA; 3 Department of Periodontics, University of Texas Health Science Center at San Antonio, San Antonio, TX 78229, USA

**Keywords:** additive manufacturing, 3D porous titanium–aluminum–vanadium, osteogenesis, Wnt16, miR-145, MSC, in vitro

## Abstract

Metal 3D printing is increasingly being used to manufacture titanium–aluminum–vanadium (Ti6Al4V) implants. In vitro studies using 2D substrates demonstrate that the osteoblastic differentiation of bone marrow stromal cells (MSCs) on Ti6Al4V surfaces, with a microscale/nanoscale surface topography that mimics an osteoclast resorption pit, involves non-canonical Wnt signaling; Wnt3a is downregulated and Wnt5a is upregulated, leading to the local production of BMP2 and semaphorin 3A (sema3A). In this study, it was examined whether the regulation of MSCs in a 3D environment occurs by a similar mechanism. Human MSCs from two different donors were cultured for 7, 14, or 21 days on porous (3D) or solid (2D) constructs fabricated by powder-bed laser fusion. mRNA and secretion of osteoblast markers, as well as factors that enhance peri-implant osteogenesis, were analyzed, with a primary focus on the Wnt family, sema3A, and microRNA-145 (miR-145) signaling pathways. MSCs exhibited greater production of osteocalcin, latent and active TGFβ1, sema3A, and Wnt16 on the 3D constructs compared to 2D, both of which had similar microscale/nanoscale surface modifications. Wnt3a was reduced on 2D constructs as a function of time; Wnt11 and Wnt5a remained elevated in the 3D and 2D cultures. To better understand the role of Wnt16, cultures were treated with rhWnt16; endogenous Wnt16 was blocked using an antibody. Wnt16 promoted proliferation and inhibited osteoblast differentiation, potentially by reducing production of BMP2 and BMP4. Wnt16 expression was reduced by exogenous Wnt16 in 3D cells. Addition of the anti-Wnt16 antibody to the cultures reversed the effects of exogenous Wnt16, indicating an autocrine mechanism. Wnt16 increased miR-145-5p, suggesting a potential feedback mechanism. The miR-145-5p mimic increased Wnt16 production and inhibited sema3A in a 3D porous substrate-specific manner. Wnt16 did not affect sema3A production, but it was reduced by miR-145-5p mimic on the 3D constructs and stimulated by miR-145-5p inhibitor. Media from 7-, 14-, and 21-day cultures of MSCs grown on 3D constructs inhibited osteoclast activity to a greater extent than media from the 2D cultures. The findings present a significant step towards understanding the complex molecular interplay that occurs in 3D Ti6Al4V constructs fabricated by additive manufacturing. In addition to enhancing osteogenesis, the 3D porous biomimetic structure inhibits osteoclast activities, indicating its role in modulating bone remodeling processes. Our data suggest that the pathway mediated by sema3A/Wnt16/miR145-5p was enhanced by the 3D surface and contributes to bone regeneration in the 3D implants. This comprehensive exploration contributes valuable insights to guide future strategies in implant design, customization, and ultimately aims at improving clinical outcomes and successful osseointegration.

## 1. Introduction

Wnt family proteins play a major role in the osteoblast differentiation of bone marrow stromal cells (MSCs). They function as upstream regulators of bone morphogenetic proteins (BMPs), which induce osteoblast differentiation and bone formation [1]. Canonical Wnt signaling proteins like Wnt3a and β-catenin are upregulated during the differentiation of MSCs on tissue culture polystyrene (TCPS) in osteogenic media (OM) [2]. However, on microroughened titanium and titanium–aluminum–vanadium (Ti6Al4V) substrates, non-canonical Wnt signaling is a critical regulatory pathway in this differentiation process, particularly in bone formation adjacent to a Ti or Ti alloy implant surface. When MSCs are cultured on Ti or Ti6Al4V substrates with surfaces that mimic an osteoclast resorption pit on bone, their integrin expression shifts from alpha5, beta1 (α5β1) to α2β1 and there is a corresponding shift in Wnt signaling from Wnt3a to non-canonical Wnt5a [3,4]. This is accompanied by a shift in shape from mesenchymal to osteoblastic [5], and the production of proteins associated with an osteoblast phenotype, including osteocalcin, osteopontin, and osteoprotegerin. Factors that promote osteoblast differentiation and osteogenesis are also upregulated, including RUNX2, BMP2, BMP4, semaphorin 3A (sema3A), and VEGF-165 [6], as well as receptors for BMP2 and VEGF-165. Subsequent studies have shown that the shift from canonical to non-canonical Wnt signaling is mediated by non-canonical Wnt11 [7], which is key to the change in planar cell polarity, including cytoskeletal polymerization and cell shape. This non-canonical signaling pathway takes precedence in cells responding to microroughened Ti surfaces [7,8].

Non-canonical Wnt5a, Wnt11, and Wnt16 are upregulated in MSCs cultured on Ti surfaces with microscale/nanoscale topography and hydrophilic chemistry [9]. The relationship of Wnt16 to other Wnts impacted by surface topography and/or chemistry is not known. Wnt16 plays a role in cortical bone stability [10]. Mouse models, with either over-expression or deletion of Wnt16, displayed increased or reduced cortical bone thickness, respectively [11,12,13]. These findings suggest that Wnt16 may be involved in mechanisms that modulate implant osseointegration, contributing to implant stability and retention via modulation of the cortical bone.

MicroRNAs are short non-coding RNA molecules that regulate mRNA activities within the cell. Studies have shown that Sema3A production may be affected by microRNA-145-5p (miRNA145-5p) and regulate bone formation and resorption [14,15]. Sema3A is suppressed by miRNA-145-5p, which suppressed the osteogenic differentiation of adipose-derived stem cells. Since Wnt16 is also involved in bone regulation, we examined the potential for miRNA145-5p to modulate the action of Wnt16 in bone. Moreover, communication between cells within the peri-implant space can be affected, including modulation of osteoclastic bone resorption and remodeling, which are critical for the successful osseointegration of implants in cortical bone as found in implant inserts in osteoporosis [16,17].

Traditionally, the studies examining osseointegration have used machined implants that present a two-dimensional (2D) surface to surrounding bone. However, we recently showed that enhanced osseointegration, including cortical bone stability, is attained with the use of trabecular bone-like three-dimensional (3D) porous Ti6Al4V implants inserted in long bone [18], or as an onlay on the calvaria [19]. Taken together, these findings suggest that 3D porous Ti alloy surfaces enhance osteogenesis through regulatory pathways comparable to those that mediate osteoblast differentiation on fully dense 2D substrates, with a specific emphasis on the non-canonical Wnt family member, Wnt16. The goal of this study was to unravel the underlying mechanisms involved in the response of MSCs to 3D porous implants, providing insights into the interplay of local factors in osseointegration. Understanding these regulatory landscapes inspires future strategies for implant design and customization, ultimately contributing to improved clinical outcomes and successful osseointegration.

We took advantage of additive manufacturing (AM), also known as 3D printing, to fabricate the implants we used in this study. We have used this method to produce constructs and implants that have a porous structure based on the trabecular morphology of the proximal femur of human bone [20]. The surface of the constructs is modified by grit blasting and acid etching using the same parameters used to modify the surface of fully dense 2D implants. We have shown that MSCs differentiate into osteoblasts when cultured on these 3D constructs [20,21] and they are well integrated with cortical bone when implanted in long bone defects [18] and as a block inserted in the calvaria [19], making them an excellent model for testing our hypothesis.

## 2. Materials and Methods

### 2.1. Construct Manufacturing

#### 2.1.1. Computer-Aided Design of Constructs

Following the methodology outlined previously [21], constructs were created using trabecular bone as the biomimetic template. A human femoral head, retrieved from a hip replacement, underwent a computed tomographic (CT) scan using a μCT 40 scanner from Scanco Medical with a voxel size of 16 μm. Subsequently, the biomimetic template was generated using Scanco software Version 7.10.1.1, undergoing 24 rotations and superimpositions (Supplementary Figure S1). The geometric design was replicated using Materialize Magics in Leuven, Belgium. Finally, a 3D rendering of this geometry as well as a fully dense version (2D) were manufactured into circular discs made of Ti6Al4V, each measuring 15 mm in diameter and 6 mm in height, suitable for placement in a 24-cell culture well plate (Supplementary Figure S1).

#### 2.1.2. Surface Treatment

Implants that undergo no further post-build modifications may exhibit partially sintered particles on their surfaces. The presence of these residual microparticles poses a risk of detachment during implant placement, and small metal particulates have known cytotoxic effects [22]. To address this concern, the constructs underwent grit-blasting and acid-etching procedures post-build, following the methods detailed in previous studies [20,21]. Briefly, both solid and porous constructs were initially grit-blasted with calcium phosphate using proprietary technology from AB Dental in Ashdod, Israel. Subsequently, the constructs underwent acid-etching through ultrasonication in a 0.3 N nitric acid solution for 5 min at 45 °C, followed by two rinses in ultrapure water at 25 °C for 5 min each. Further rinsing in 97% methanol for 5 min preceded pickling to eliminate impurities. The pickling treatment involved three 10 min ultrasonic rinses in ultrapure distilled H_2_O, followed by submersion in a 1:1 solution of 20 g/L NaOH and 20 g/L H_2_O_2_ for 30 min at 80 °C, and ultrasonication in ultrapure distilled H_2_O for 10 min. The constructs were then degreased for 12 min, submerged in 65% aqueous nitric acid at 100 °C for 10 min, and finally rinsed three times in ultrapure distilled H_2_O for 10 min each. The constructs were blotted, air-dried, and sterilized using gamma irradiation before undergoing analysis and subsequent cell culture.

### 2.2. Material Characterization

#### 2.2.1. Scanning Electron Microscopy (SEM)

Surface topography was examined by SEM using a Hitachi SU-70 instrument (Tokyo, Japan). Constructs were affixed securely to SEM imaging mounts using carbon tape. The imaging process employed a 56 μA ion current, 5 kV accelerating voltage, and maintained a 5 mm working distance. A comprehensive evaluation was ensured by imaging ten locations per disk at each magnification level, with a minimum of two disks per group subjected to imaging procedures.

#### 2.2.2. Laser Confocal Microscopy

For the qualitative assessment of surface roughness, laser confocal microscopy (LCM) wasemployed, utilizing a Zeiss LSM 710 instrument. Z-stacks were acquired using a PlanApochromat 20×/0.8 M27 objective with a ×5 optical zoom, employing a 405 nm laser in reflection mode at 50% power. The scanning parameters included a 0.39 μs pixel dwell, 25 μm pinhole, 85.02 × 85.02 μm image size, and a step size of 1 μm. The average surface microroughness parameter (Sa) was evaluated, defined as the average absolute distance in the z-plane. The values for the parameter were obtained using ZEN software from Zeiss and are presented as the mean and standard error for two samples per group, with three measurements per sample.

#### 2.2.3. Micro-Computed Tomography

To analyze construct properties, micro-computed tomography (micro-CT) using a Skyscan 1173 system (Bruker, Kontich, Belgium) was employed. The 3D constructs underwent scanning at a resolution of 1120 × 1120 pixels. The scanning process used a brass 0.25 mm filter, operated at a voltage of 100 kV, a current of 80 μA, and employed an image pixel scale of 15.1 μm. Additional parameters included an exposure time of 250 ms and a rotation step of 0.2 degrees. For the reconstruction process, a standard Feldkamp reconstruction was conducted with a Gaussian smoothing kernel set to zero, and a beam hardening correction of 20%, using NRecon software version 1.6.9.17 from Bruker. The analysis was performed using CT-Analyser version 1.14.4.1 (Bruker). The constructs were binarized, and various properties such as construct volume, strut thickness, and separation were calculated within a fixed volume of interest, with values averaged for three constructs per group.

### 2.3. Cell Culture

#### 2.3.1. Bone Marrow Stromal Cells

Human bone MSCs were purchased from Ossium Health (Indianapolis, IN, USA). Donor 1 (MSC127) cells were sourced from a white, 23-year-old female; donor 2 (SC003) cells were sourced from a Hispanic, 16-year-old male donor. Cells were cultured in growth media (GM), consisting of Alpha-modified Eagle medium (αMEM, Life Technologies, Carlsbad, CA, USA), 2 mM L-glutamine, and 10% heat-inactivated fetal bovine serum (HI FBS) (Gemini Bioscience, Calabasas, CA, USA), along with 50 U/mL penicillin-50 µg/mL streptomycin (VWR International, Radnor, PA, USA). Cultures were maintained at 37 °C in 5% CO_2_ and 100% humidity. The cell densities plated are described under each section.

#### 2.3.2. Human Osteoclast Precursors (OCPs)

Cultivation of human osteoclast precursors (Lonza Biosciences, Walkersville, MD, USA) at 10,000 cells/well involved placing them on a 96-well OsteoLyse^TM^ Assay Kit coated with human collagen type I for seven days [23]. Cells were cultured in osteoclast precursor growth medium (OCGM, Lonza Biosciences), which was supplemented with 33 ng/mL macrophage colony-stimulating factor (M-CSF, Lonza Biosciences) and 66 ng/mL receptor activator of nuclear factor kappa-Β ligand (RANKL). In addition to the standard culture, osteoclast precursors were also cultured in OCGM supplemented with 33 ng/mL M-CSF, excluding the addition of RANKL. This served as the negative control for the experimental setup.

### 2.4. Biological Responses

#### 2.4.1. Cellular Responses on 3D Porous and Fully Dense Ti6Al4V Disks

MSCs were plated on 3D and 2D additively manufactured disks at a density of 30,000 cells/cm^2^. Cells were cultured at the same density on tissue culture polystyrene (TCPS) as a control. Incubation occurred at 37 °C, 5% CO_2_, and 100% humidity throughout the entire experimental period. Media changes were initiated 24 h after plating and subsequently every 48 h. At intervals of 7, 14, or 21 days, fresh media were added to the cultures and the media that now contained any proteins synthesized or metabolized by the cells as well as lipids, ions, and other small molecules (conditioned media, CM) collected 24 h later and stored at −80 °C. Osteocalcin (OCN, R&D Systems, Minneapolis, MN, USA), BMP2 (R&D Systems), BMP4 (R&D Systems), sema3A (Lifespan Biosciences, Seattle, WA, USA), OPG (R&D System), Wnt3a (Biomatik, Kitchener, ON, Canada), Wnt11 (Biomatik), Wnt5a (Biomatik), and Wnt16 (Biomatik) were measured in the conditioned media by ELISA following the manufacturer’s directions. An aliquot of the conditioned media was removed and assayed without prior acidification to determine the levels of active TGF-β1 using an immunoassay kit from R&D Systems. Total TGF-β1 was determined following acid activation of a second aliquot of conditioned media. Latent TGF-β1 was assessed by subtracting active growth factor from the total amount. The QuantiFluor dsDNA system (Promega, Madison, WI, USA) was used to determine the total DNA content in cell layer lysates by fluorescence. All protein levels were normalized to total DNA content. Additionally, conditioned media from MSCs on day 7, day 14, and day 21 were collected specifically for treating human OCPs, as outlined in Section 2.4.3. Alkaline phosphatase-specific activity in the lysates was assayed by measuring the release of *p*-nitrophenol from *p*-nitrophenyl phosphate at a pH of 10.2, and results were normalized to protein content of the cell lysates.

#### 2.4.2. Autocrine Effect of Wnt16 on Cellular Response to Ti6Al4V Constructs

To investigate whether the cellular response to Ti4Al6V is influenced by Wnt16, we first examined the effects of exogenous recombinant human Wnt16 (rhWnt16, R&D Systems). MSCs were plated as described above. The cultures underwent continuous treatment with 100 ng/mL rhWnt16 at each media change. On day 7, MSCs received fresh media without rhWnt16 for 24 h, after which the CM were collected for the measurement of local factor production. To explore autocrine regulation by endogenous Wnt16, hBMSCs were treated with media containing 2 μg/mL rabbit polyclonal anti-human Wnt16 antibody (ThermoFisher Scientific, Waltham, MA, USA) or human IgG isotype control antibody (ThermoFisher Scientific) as IgG controls. This treatment was conducted using the same experimental setup as rhWnt16 treatment. ELISAs were used to determine the levels of osteogenic factors and local factors in the CM, as follows: OCN, OPN, BMP2, BMP4, VEGF-165 (VEGF-A), sema3A (Lifespan Biosciences), OPG, Wnt3a, Wnt11, Wnt5a, and Wnt16.

The same experimental setup was repeated for measuring Wnt16 and miR-145-5p expression. After 7 days of rhWnt16 treatment, MSCs were cultured with fresh media without rhWnt16 for 12 h and mRNA was harvested as described below. mRNA was extracted from cells using TRIzol (Invitrogen, Carlsbad, CA, USA); RNA was quantified using a NanoDrop spectrophotometer (ThermoFisher) and then reverse-transcribed into cDNA. Real-time PCR was performed using a fluorescent dye (Power SYBR Green, Applied Biosystems, Foster City, CA, USA) to quantify starting mRNA levels using gene-specific primers, including glyceraldehyde 3-phosphate dehydrogenase (GAPDH), Wnt16, and miR-145 (Table 1). The normalization was performed by evaluating fold regulation values (∆∆Ct), which were determined by normalizing CT values to GAPDH cycle values (∆Ct), and then again to the TCPS control cycle (∆∆Ct) values. After normalization, a fold change of 2 was determined to be significant based on *p* < 0.05.

#### 2.4.3. Paracrine Effect of the 3D Construct on Osteoclast Activity

MSCs were cultured on TCPS, solid Ti6Al4V, and porous Ti6Al4V for 7, 14, or 21 days, as described above. Simultaneously, OCPs were cultured using the OsteoLyse^TM^ Assay Kit [24]. After a 7-day culture period, 200 µL of CM from hBMSCs were used to treat differentiated OCPs on the OsteoLyse^TM^ Assay Kit for 2 days. Each experiment incorporated positive controls, where OCPs received OCGM supplemented with 33 ng/mL M-CSF and 66 ng/mL RANKL, as well as negative controls, where OCPs received OCGM supplemented with 33 ng/mL M-CSF but not RANKL. Osteoclast activity was assessed by measuring the release of europium-conjugated human type I collagen-coated on the bottom of the OsteoLyse Assay Kit at various time points. For this, 200 µL of a fluorophore-releasing reagent (Lonza Biosciences) was added to each well of a 96-well black, clear-bottom assay plate (Corning Inc., Corning, NY, USA). Subsequently, 10 µL of cell culture supernatant was transferred to each well containing the fluorophore-releasing reagent. The fluorescence of each well was measured using an excitation wavelength of 340 nm and an emission wavelength of 615 nm over a 400 µs period following an initial delay of 400 µs.

#### 2.4.4. The Effect of miR-145-5p on Differentiation of MSCs

MSCs were cultured in control media or differentiation media with or without miR-145-5p mimic (Thermo Fisher Scientific Inc., Waltham, MA, USA, mirVana #4464066) or with the miR-145-5p inhibitor (Thermo Fisher Scientific, mirVana #4464084). For each experiment, there were six independent wells per variable (control media, differentiation media, 20 nM miR-145-5p mimic or inhibitor + control media, 20 nM miR-145-5p mimic/inhibitor + differentiation media, 20 nM miR-145-5p mimic/inhibitor + control media, 40 nM miR-145-5p mimic/inhibitor + differentiation media). Cells were allowed to attach for 24 h following plating before media were changed and then every 48 h thereafter. Control media were identical to GM described above (αMEM, Life Technologies, Carlsbad, CA, USA), 2 mM L-glutamine, and 10% heat-inactivated fetal bovine serum (HI FBS, Gemini Bioscience in Calabasas, CA, USA), along with 50 U/mL penicillin-50 µg/mL streptomycin (VWR International, Radnor, PA, USA). Differentiation media were chemically comparable to osteogenic media (OM), consisting of GM supplemented with 10 mM β-glycerophosphate, 50 µg/mL ascorbic acid, and 10^−8^ M dexamethasone. On day 11, cells were transfected with miR-145-5p mimic or inhibitor using lipofectamine as the vehicle. Control groups received empty lipofectamine. On day 13, cells were incubated in fresh media for 24 h before harvesting. CM were collected and immediately stored at −80 °C. Cell layer lysates were rinsed twice with 1 mL 1× PBS, lysed in 0.5 mL 0.5% Triton X-100, and immediately stored at −80 °C for biological assays. Cell response was evaluated as previously described [11,13]. In brief, cell layers were suspended in 0.5% Triton X-100 and lysed by ultra-sonification at 40 V for 10 s/well (VCX 130; Vibra-Cell, Newtown, CT, USA). Total DNA content was measured using the QuantiFluor dsDNA system (Promega, Madison, WI, USA). Alkaline phosphatase specific activity (ALP) in the lysates was assayed by measuring the release of *p*-nitrophenol from *p*-nitrophenyl phosphate at a pH of 10.2 and normalized to total protein content in the cell lysate. ELISAs were performed to determine levels of osteogenic markers. OCN (DY141905), and OPN (DY1433) were quantified according to the manufacturer’s protocols (R&D Systems, Inc.). Production of proteins was normalized to total DNA content.

#### 2.4.5. Regulation of Wnt16 Production on Porous Ti6Al4V

To determine if miR-145-5p plays a role in Wnt16 production on porous Ti4Al6V, MSCs were cultured on 3D constructs at 15,000 cells/cm^2^. At 60% confluence, MSCs were transfected for 24 h with 20 nM of mirVana miRNA mimic using 0.2% lipofectamine RNAimax (Lipo; Life Technologies) in antibiotic-free CCM. Lipo groups served as a negative control. Following mimic transfection, fresh media were added to the cells and cells were harvested after 24 h as described above to measure Wnt16 and sema3A protein production.

### 2.5. Statistical Analysis

Data are presented as means ± standard error mean for the number of sample points described in the material characterization. Data are presented as means ± standard error mean of six independent cultures/variable; in vitro cell experiments were repeated to ensure validity. A two-tailed *t*-test was used to determine significant differences for experiments with only two groups. Experiments with more than two groups were subjected to a one-way analysis of variance with a two-tailed Tukey correction to adjust for multiple comparisons to maintain an experiment-wide error rate (α) of 0.05. All statistical analyses were performed using JMP statistical software - Pro 17 (SAS Institute Inc., Cary, NC, USA), and all graphs were made using GraphPad Prism 10.1 (GraphPad, La Jolla, CA, USA).

## 3. Results

### 3.1. Solid and Porous Ti6Al4V Constructs Have Comparable Surface Topography

**Figure 1:**
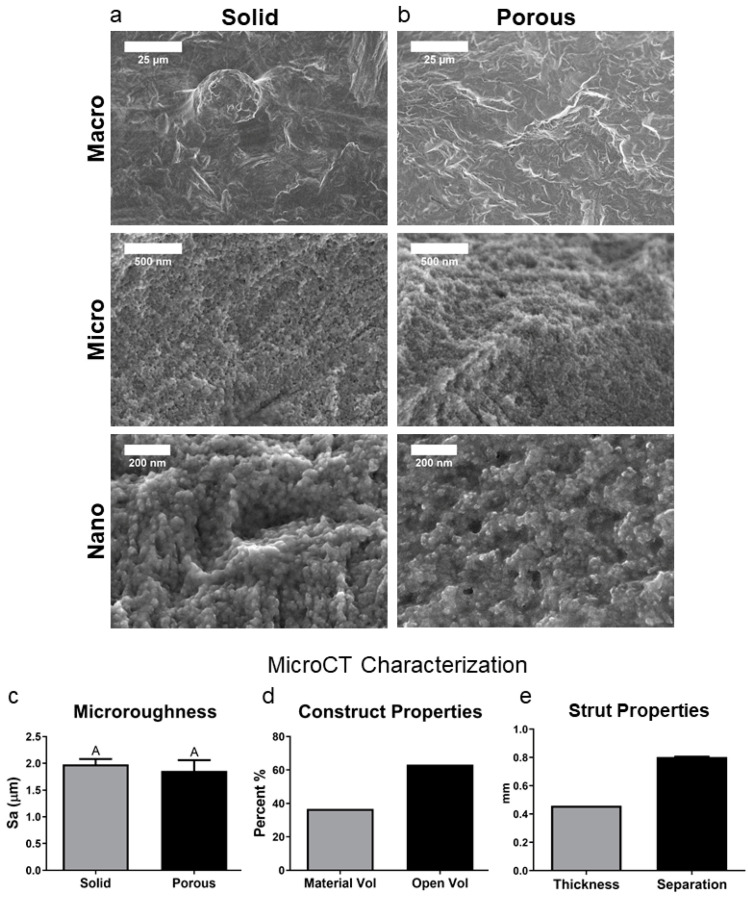
Construct characterization. (**a**) SEM of a solid construct at macro, micro, and nano levels; (**b**) SEM of a porous construct at macro, micro, and nanoscale; (**c**) surface roughness characterization by laser confocal microscopy is expressed as Sa; (**d**) micro CT measurement of the % volume of the implant represented by metal vs. the open volume of the implant; (**e**). micro CT assessment of the thickness and separation of struts in the porous constructs measured by distance in mm. Groups not sharing the same letters are significant at *p* < 0.05.

The topological characteristics of both solid and 3D constructs were evaluated using SEM to discern roughness at macro (25 μm), micro (500 nm), and nano (200 nm) scales revealed strikingly similar surface topography on both constructs (Figure 1a,b). This similarity was confirmed by microroughness quantification through confocal laser microscopy (Figure 1c). The 3D constructs had a volume percentage of approximately 38%, with an open volume percentage of around 62% (Figure 1d). The strut thickness of the porous 3D constructs was 0.42 mm, with a separation of struts at 0.8 mm (Figure 1e). These results are in agreement with our previous full characterization of these surfaces [24].

### 3.2. Local Factor Production Varies with Construct Type and Incubation Time

**Figure 2:**
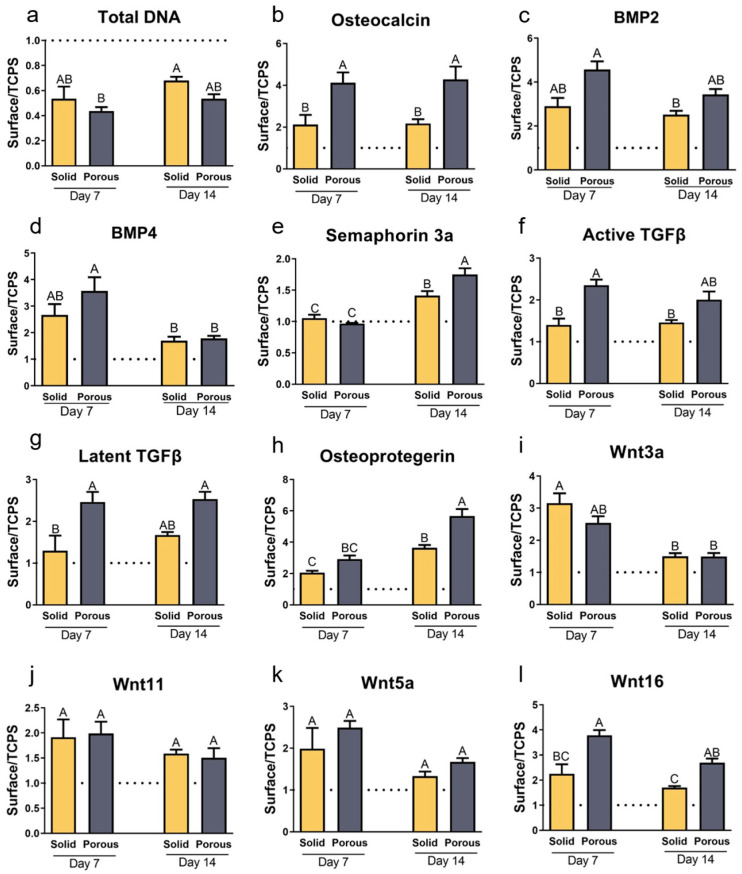
Response of human bone MSCs to the solid and porous constructs at 7 and 14 days of culture. (**a**) Total DNA content was determined in the cell layer lysate. (**b**) Osteocalcin, (**c**) bone morphogenetic protein 2 (BMP2), (**d**) BMP 4, (**e**) semaphorin 3A, (**f**) active and (**g**) latent transforming growth factor beta 1, (**h**) osteoprotegerin, (**i**) Wnt3a, (**j**) Wnt11, (**k**) Wnt5a, and (**l**) Wnt16 were determined in the conditioned media after 24 h. The data are presented as the ratio of values in the conditioned media of cells on the solid or porous surface compared to cultures grown on tissue culture polystyrene (TCPS). Data from one of at least two experiments are presented. Data are the means ± SEM, for N = 6 independent cultures per variable. Groups not sharing the same letters are significant at *p* < 0.05.

MSCs cultured on each substrate have a similar morphology, with long denticles attached to the rough surface (Supplementary Figure S2). Compared to TCPS, the DNA content of MSCs grown on both constructs was reduced and to a comparable extent (Figure 2a and Supplementary Figure S3a). While osteocalcin production was increased by 100% compared to TCPS, it was increased by 400% on the porous constructs by 7 days and remained elevated at 14 days (Figure 2b and Supplementary Figure S3b), but by 21 days, the osteocalcin content of the conditioned media in the 3D cultures was reduced to the level in the 2D cultures (Supplementary Figure S3b). Osteopontin production on the Ti6Al4V constructs was higher than on TCPS at all time points, and on day 14, levels in the 3D cultures were more than doubled compared to the 2D cultures (Supplementary Figure S3c). BMP2 and BMP4 levels were elevated compared to TCPS at day 7 and 14, with no difference as a function of construct porosity (Figure 2c,d). BMP2 was reduced at day 21 compared to day 14 on the porous constructs (Supplementary Figure S3d). Production of sema3A was increased on both constructs at 14 days compared to TCPS, with the greatest increase in the 3D cultures (Figure 2e). Active TGFβ1 and latent TGFβ1 were elevated on both constructs by day 7, but the increase was greatest in the 3D cultures (Figure 2f,g).

Osteoprotegerin production was elevated by day 7, but by day 14, the increase in OPG was twice as much as on day 7 in the 2D cultures and 3 times as much in the 3D cultures (Figure 2h). Osteoprotegerin remained constant in the 2D cultures over time whereas by day 21, production was 15-fold greater in the 3D cultures (Supplementary Figure S3e). This marked increase in VEGF production on day 21 was mirrored in the porous Ti6Al4V cultures (Supplementary Figure S3f).

Levels of Wnt family proteins in the conditioned media were also sensitive to the porosity of the constructs. Wnt3a was markedly elevated at day 7 in both 3D and 2D cultures compared to TCPS; levels were reduced by day 14 to a comparable extent in the cultures (Figure 2i). Wnt11 and Wnt5 were increased on days 7 and 14 to a comparable extent, with no difference as a function of construct porosity (Figure 2j,k). In contrast, Wnt16 production was increased compared to TCPS, and this increase was greater in the 3D cultures on both days (Figure 2l).

MSCs from donor 2 exhibited comparable responses to the Ti6Al4V constructs (Supplementary Figure S4). DNA content was reduced on day 14 to a greater extent in the porous cultures (Supplementary Figure S4a,m). Alkaline phosphatase specific activity was reduced compared to TCPS by day 7 and the reduction was greatest in the 3D cultures (Supplementary Figure S4b,n). In contrast, osteocalcin production was increased on both Ti6Al4V constructs, with the greatest increase on the 3D constructs at both time points (Supplementary Figure S4c,o). Donor 2 cells exhibited a marked increase in BMP2 production by day 7 in 2D and 3D cultures; by day 14, the effect of the porous construct on BMP2 production was greater (Supplementary Figure S4d,p). BMP4 levels were increased in the 3D cultures at day 7; by day 14, BMP4 was increased in cultures grown on both Ti6Al4V constructs (Supplementary Figure S4q). There was no change in sema3A in 7-day cultures grown on either construct, but by day 14, sema3A production was increased on 2D cultures and to a greater extent on 3D cultures (Supplementary Figure S4f,r). Donor 2 MSCs exhibited increased active TGFβ1 production at 7 and 14 days, with the greatest increase in 3D cultures (Supplementary Figure S4h,s). There was a 2-fold increase in latent TGFβ1 on both Ti6Al4V constructs at 7 days; by day 14, production was greater in the 3D cultures (Supplementary Figure S4h,t). Donor 2 MSCs exhibited a greater increase in VEGF production in 2D cultures than was seen in Donor 1 cultures at 7 days; in both cases, the VEGF production at day 14 was significantly greater on the porous constructs (Supplementary Figure S4i,u). Wnt11 levels in the 2D and 3D cultures were increased over TCPS to a comparable extent on day 7 and 14 (Supplementary Figure S4j,v). Wnt5a was also increased at day 7 on the solid and porous Ti4Al6V compared to TCPS, but there were no statistically significant differences in Wnt5a on day 14 (Supplementary Figure S4k,w). Wnt16 production was elevated compared to TCPS by day 7 in both 2D and 3D cultures. The increase was greater on the porous constructs and this substrate specific differential was retained at day 14 (Supplementary Figure S4l,x).

### 3.3. Wnt16 Increased Cell Proliferation and Inhibited the Impact of 3D Porosity on Osteoblast Differentiation

**Figure 3:**
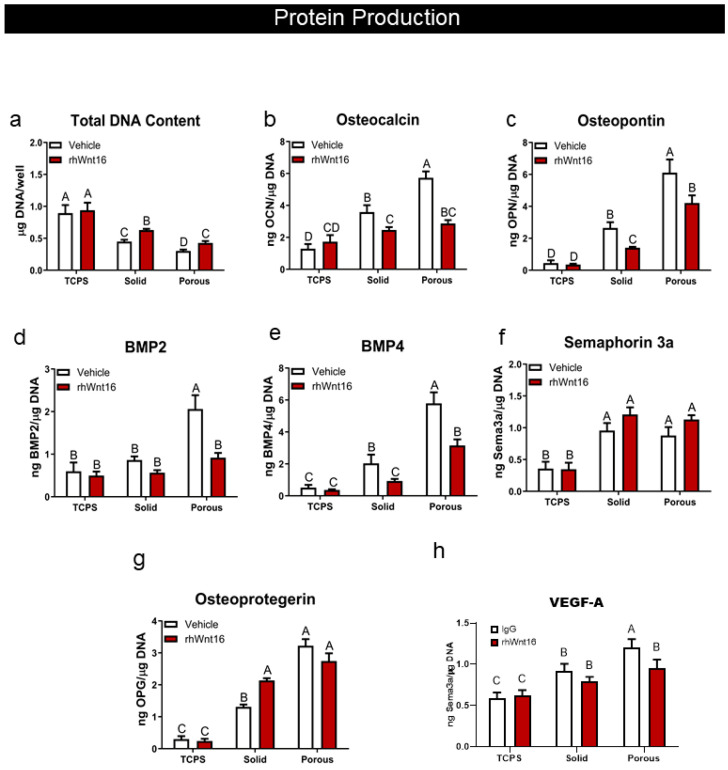
Effect of 100 ng/mL rhWnt16 on protein production by human bone MSCs grown on solid and porous Ti6Al4V constructs after 7 days of culture in growth media. Cells cultured on TCPS served as controls. (**a**) Total DNA content was determined in the cell layer lysate. (**b**) Osteocalcin, (**c**) osteopontin, (**d**) bone morphogenetic protein 2 (BMP2), (**e**) BMP 4, (**f**) semaphorin 3A, (**g**) osteoprotegerin, and (**h**) vascular endothelial growth factor A were determined in the conditioned media after 24 h. Data were normalized to the DNA content of the cultures. Data from one of at least two experiments are presented. Data are the means ± SEM, for N = 6 independent cultures per variable. Groups not sharing the same letters are significant at *p* < 0.05.

Wnt16 had no impact on the DNA content of cultures grown on TCPS (Figure 3a). However, it reduced the inhibitory effect of the 2D and 3D constructs on DNA content. In contrast, it reduced the stimulatory effect of the Ti6Al4V constructs on osteocalcin (Figure 3b) and osteopontin (Figure 3c), blocking the osteocalcin increase on 3D constructs and significantly reducing the increase in osteopontin on the porous constructs. Similarly, Wnt16 blocked the increase in BMP2 and BMP4 associated with the 3D constructs (Figure 3d,e). Wnt16 did not affect sema3A production on any surface (Figure 3f). It caused an increase in osteoprotegerin on solid Ti6Al4V constructs to levels observed in 3D cultures, but it did not cause a further increase on the porous constructs (Figure 3g). Wnt16 blocked the increase in VEGF-A associated with the 3D constructs (Figure 3h).

**Figure 4:**
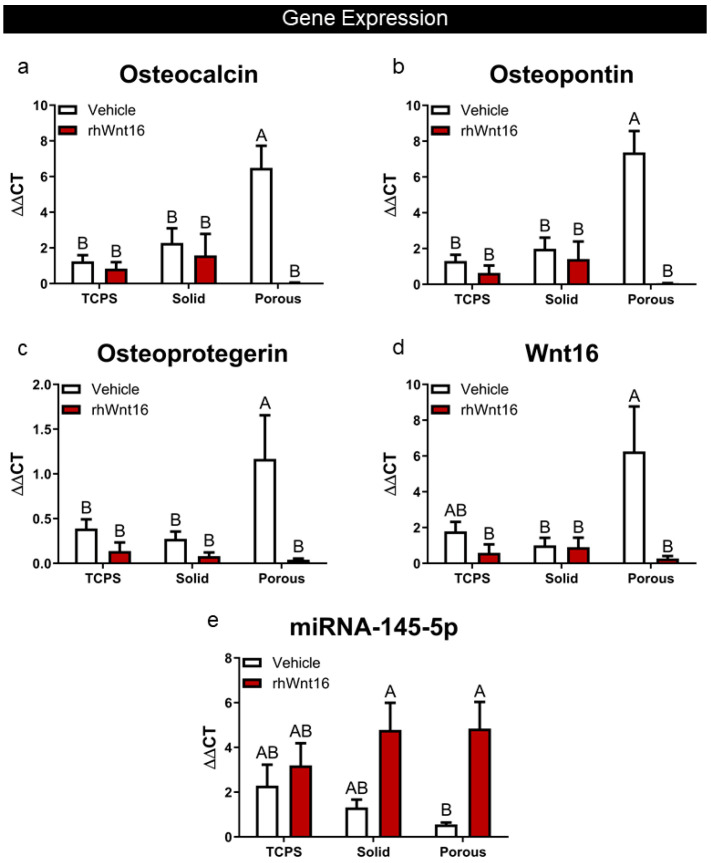
Effect of 100 ng/mL rhWnt16 on the expression of (**a**) osteocalcin, (**b**) osteopontin, (**c**) osteoprotegerin, (**d**) Wnt16, and (**e**) miRNA-145-5p in human bone MSCs cultured for 7 days in growth media on TCPS, solid Ti6Al4V constructs, or porous Ti6Al4V constructs. Data from one of at least two experiments are presented. Data are the means ± SEM, for N = 6 independent cultures per variable. Groups not sharing the same letters are significant at *p* < 0.05.

The effect of Wnt16 was at the gene expression level. In cultures grown on the 3D constructs, treatment with Wnt16 reduced mRNAs for osteocalcin, osteopontin, and osteoprotegerin (Figure 4a–c). Exogenous rhWnt16 also blocked endogenous Wnt16 expression (Figure 4d). However, miRNA-145-5p was upregulated in the 3D cultures (Figure 4e).

**Figure 5:**
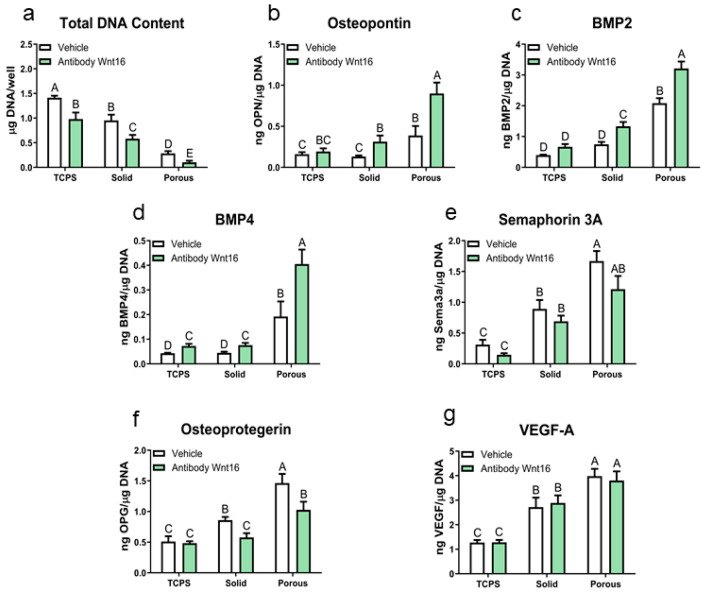
Effect of anti-rhWnt16 antibodies on protein production by human bone MSCs grown on solid and porous Ti6Al4V constructs after 7 days of culture in growth media. Cells cultured on TCPS served as controls. (**a**) Total DNA content was determined in the cell layer lysate. (**b**) Osteopontin, (**c**) BMP2, (**d**) BMP 4, (**e**) semaphorin 3A, (**f**) osteoprotegerin, and (**g**) vascular endothelial growth factor A were determined in the conditioned media after 24 h. Data were normalized to the DNA content of the cultures. Data from one of at least two experiments are presented, all showing comparable results. Data are the means ± SEM, for N = 6 independent cultures per variable. Groups not sharing the same letters are significant at *p* < 0.05.

Blocking endogenous Wnt16 with the anti-Wnt16 antibody had the opposite effects on the MSCs. Total DNA content was reduced on all surfaces, including TCPS (Figure 5a). Osteopontin and BMP2 were increased on the Ti6Al4V surfaces (Figure 5b,c) and BMP4 was increased on all surfaces (Figure 5d). Blocking endogenous Wnt16 did not alter sema3A production (Figure 5e), but it did reduce osteoprotegerin in the 2D and 3D cultures (Figure 5f). Finally, the production of VEGF was not altered by blocking endogenous Wnt16 (Figure 5g).

### 3.4. MSCs Grown on 3D Constructs Produce Factors That Inhibit Osteoclast Activity

**Figure 6:**
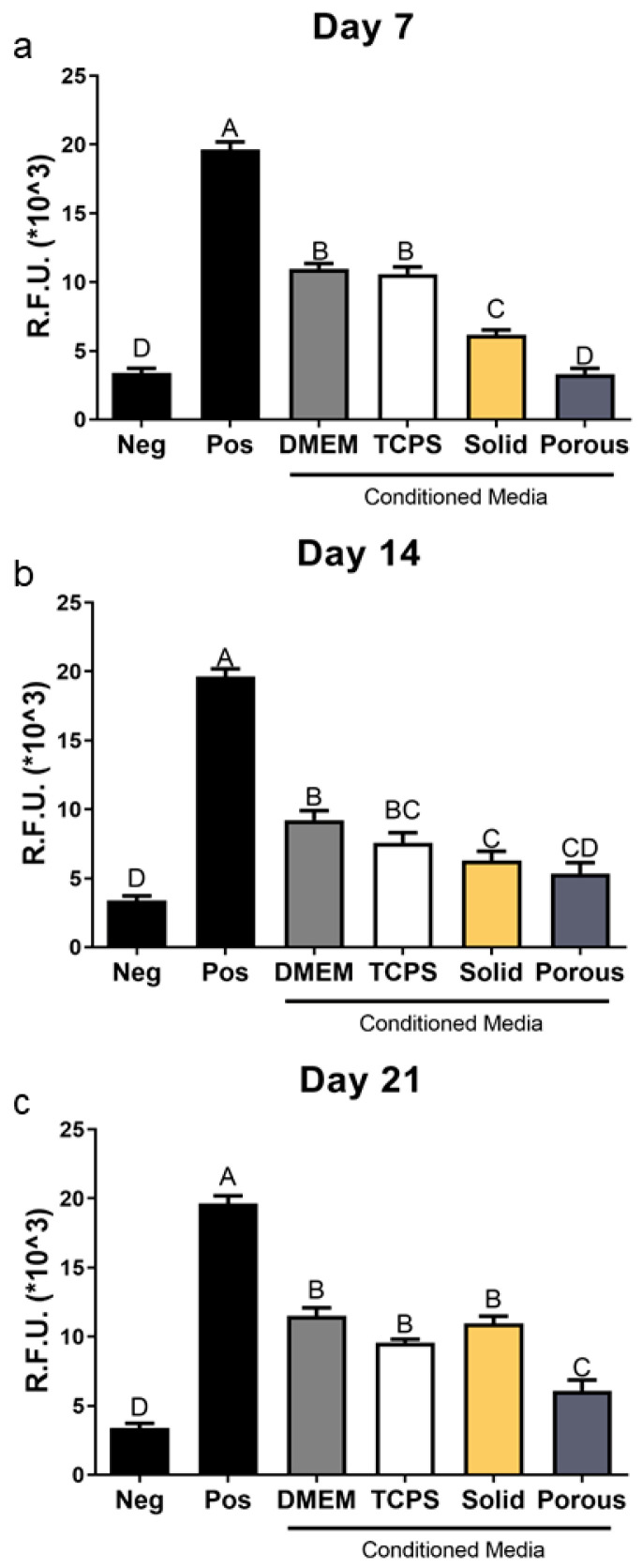
Regulation of osteoclast activity by conditioned media collected from human bone MSCs grown for (**a**) 7, (**b**) 14, and (**c**) 21 days of culture on TCPS, solid, and porous Ti6Al4V constructs. Data from one of at least two experiments are presented, all showing comparable results. Data are the means ± SEM, for N = 6 independent cultures per variable. Groups not sharing the same letters are significant at *p* < 0.05.

Conditioned media from MSCs cultured for 7 days on 2D and 3D constructs caused a decrease in osteoclast activity compared to conditioned media from cultures grown on TCPS and the effect of conditioned media from 3D cultures was greater than from 2D cultures (Figure 6a). This inhibitory effect was lost in the conditioned media from 14-day cultures (Figure 6b). However, cells grown for 21 days in 3D cultures generated conditioned media that caused a marked reduction in osteoclast activity compared to media from cultures grown on 2D cultures or TCPS (Figure 6c).

### 3.5. Wnt16 Acts via miR-145 and sema3A

**Figure 7:**
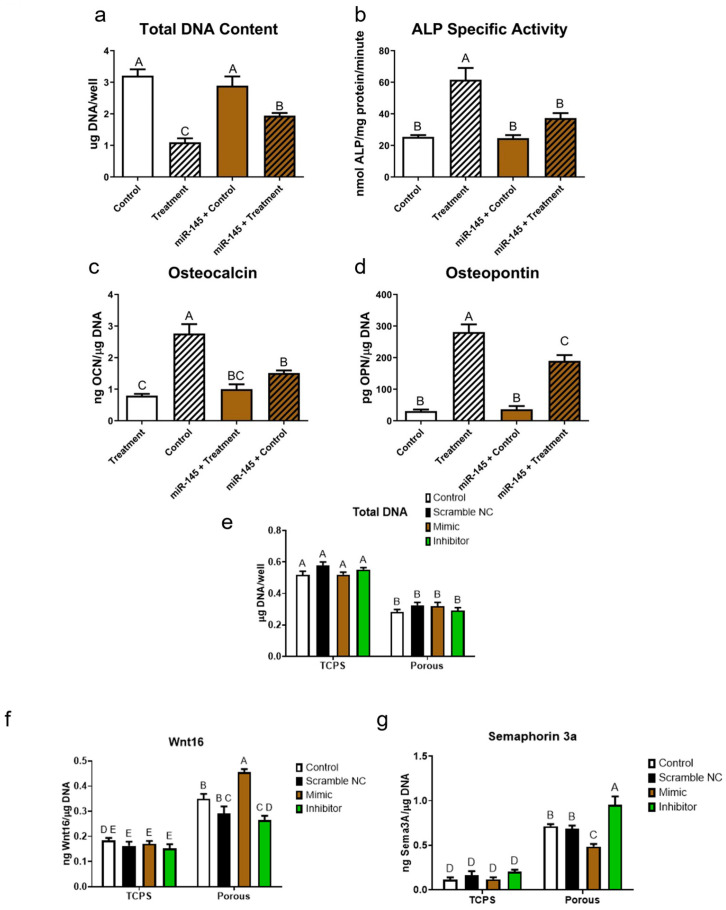
Regulation of human bone MSCs by 20 nM of miR-145. MSCs were cultured on TCPS in growth media (control) or growth media + beta-glycerol phosphate and dexamethasone (treatment). One half of each set of cultures were incubated with miR-145-5p mimic. At day 7, (**a**) total DNA and (**b**) alkaline phosphatase specific activity were measured in cell layer lysates. (**c**) Osteocalcin and (**d**) osteopontin were measured in the conditioned media. Alternatively, MSCs were cultured on TCPS or porous Ti6Al4V constructs for 11 days and transfected with lipofectamine, vehicle (negative control), miR-145-5p mimic, or miR-145-5p inhibitor. On day 13, fresh media were added to the cultures and (**e**) DNA content, (**f**) Wnt16, and (**g**) semaphorin 3A were measured 24 h later. Data from one of at least two experiments are presented, all showing comparable results. Data are the means ± SEM, for N = 6 independent cultures per variable. Groups not sharing the same letters are significant at *p* < 0.05.

miR-145 regulates osteoblast differentiation of MSCs. Osteogenic media caused a reduction in DNA content, which was reversed by treatment of the cultures with miR-145 (Figure 7a). In contrast, osteogenic media stimulated alkaline phosphatase specific activity and production of osteocalcin and osteopontin (Figure 7b–d) and this was prevented by treatment of the cultures with miR-145. The transfection of MSCs with miR-145-5p mimic or miR-145-5p inhibitor had no effect on the reduction in DNA content of cells grown on 3D cultures (Figure 7e). However, transfection with miR-145-5p mimic caused an increase in Wnt16 production compared to the lipofectamine control and miR-145-5p inhibitor caused a decrease in Wnt16 production (Figure 7f). The reverse was seen when sema3A production was measured. The miR-145-5p mimic caused a decrease and the miR-145-5p inhibitor caused an increase in sema3A production (Figure 7g).

## 4. Discussion

Our findings show that the physical environment plays a significant role in determining the fate of multipotent MSCs. In this study, MSCs exhibited an osteoblastic phenotype when cultured on a surface with a complex multiscale topography, even when grown in a media that did not contain additives normally used to induce their differentiation on TCPS. Importantly the introduction of porosity generated by additive manufacturing, using trabecular bone as the template, enhanced the surface topography effect. MSCs exhibited a mature osteoblast phenotype characterized by reduced alkaline phosphatase specific activity and increased production of osteocalcin [25]. These effects were observed at 7 days in GM, whereas MSCs do not exhibit a comparable osteoblast phenotype on TCPS unless they are cultured in osteogenic media containing beta-glycerophosphate and dexamethasone for 14 to 21 days [26]. This early expression of a well-differentiated osteoblast phenotype was due in part to the elevated production of BMP2 and BMP4, which has been noted in cultures of MSCs grown on Ti and Ti6Al4V substrates with a multiscale topography [6]. However, it is unlikely that the enhanced osteoblastic response observed on the porous scaffolds can be attributed to BMPs alone. The amount of BMP2 and BMP4 produced on the 2D and 3D constructs was comparable, suggesting that other factors played a role. For example, production of active TGFβ1, which stimulates type I collagen synthesis [27], was increased on the 3D constructs, providing a mineralizable ECM.

The osteogenic stimulus due to the porosity of the 3D constructs was enhanced in the 14-day cultures. Production of OPN, which is a multifunctional glycoprotein that aids in MSC migration and attachment [28], was increased in the 3D cultures, suggesting that it plays a role in the migration of the cells throughout the construct.

Our findings support an indirect role of the porous structure in osteogenesis by modulating bone resorption activities and blood vessel formation. We have shown previously that MSCs establish an osteoinductive microenvironment on trabecular bone-like structures [18,19,20]. In addition to secreting factors that modulate osteoblastic differentiation, MSCs generate factors that regulate the behavior of other cells in the peri-implant tissue. Semaphorin 3A, a neurotropic factor shown to stimulate osteoblast differentiation in vitro and osseointegration in vivo [29], was increased on the Ti6Al4V surfaces compared to TCPS on day 14 and further increased on the 3D constructs. Similarly, production of osteoprotegerin, a decoy receptor for RANKL [30], was elevated on day 14 on both constructs, with the greatest increase on the 3D constructs. By binding RANKL, OPG prevents activation of osteoclasts, and treatment of osteoclasts with conditioned media from the 7-day 2D and 3D cultures reduced their activity, with the greatest reduction caused by conditioned media from the 3D cultures. At 21 days, production of OPG was significantly increased in the 3D cultures, and this was correlated with a decrease in the activity of osteoclasts treated with the conditioned media from the 3D cultures.

As important as the upregulation of osteoblastic differentiation is, the regulation of osteoclastic activity is equally important in order to achieve net new bone formation in vivo. The migration of osteoprogenitor cells throughout the porosity generated by 3D printing depends in part on the ingrowth of the vasculature. Our findings show that the MSCs produce high levels of VEGF when cultured on the 3D constructs for 21 days, indicating that porosity is a variable in determining peri-implant osteogenesis.

The observation that BMP2 and BMP4, which are recognized stimulators of osteoblast differentiation, did not exhibit a significant increase during the 3D porous-mediated osteogenesis process was unexpected. Moreover, the production of upstream regulators of the BMP2 and BMP4 signaling pathway, Wnt11 and Wnt5a, remained unaffected by the introduction of the porous structure. Contrary to osteogenesis on 2D titanium surfaces, which was regulated through non-canonical Wnt11 and Wnt5a [7], this indicates that the regulatory dynamics governing these upstream regulators in 3D do not conform to traditional patterns on 2D titanium surfaces. In addition, Wnt3a, which was downregulated on 2D microtextured surfaces [2], was not regulated by the porous structure. In contrast, Wnt11, which triggers the expression of Wnt5a, and Wnt5a, which stimulate BMP2 expression and downstream osteoblast differentiation [4], were upregulated on both the 2D and 3D constructs compared to TCPS, but to a comparable extent. This suggests that the mechanisms mediating osteogenesis on the porous 3D constructs differ from those involved in osteogenesis on a 2D microtextured Ti6Al4V, and from those mediating differentiation on TCPS when cultured in osteogenic media. Our data indicate that the outcomes regulated by the sema3A/Wnt16/miRNA-145-5p pathway were enhanced on the 3D surface and made the difference in peri-implant bone regeneration observed on the 3D implants.

Unlike the production of Wnt3a, Wnt5a, and Wnt11, production of the non-canonical Wnt16 was increased on the porous constructs at both 7 and 14 days, compared to cultures grown on TCPS and solid Ti6Al4V. This suggested the possibility that Wnt16 acted to stimulate osteogenesis in the 3D cultures. However, treatment of MSCs on the porous constructs with rhWnt16 blocked the expression of mRNAs for osteocalcin, osteopontin, and osteoprotegerin and significantly reduced their levels in the conditioned media. Exogenous Wnt16 also blocked the expression of Wnt16 mRNA, suggesting that endogenous Wnt16 might be involved. This proved to be the case. Treatment of the cultures with antibodies to Wnt16 caused an increase in osteopontin, BMP2 and BMP4 on both Ti6Al4V constructs with the greatest effect in the 3D cultures. At the same time, blocking endogenous Wnt16 reduced the production of osteoprotegerin.

MSCs produced increased levels of sema3A when cultured for 14 days on the Ti6Al4V surfaces and this was further enhanced on the 3D constructs without a similar increase in BMP, raising the question of the role of sema3A in osteogenesis in 3D constructs. In other studies, sema3A stimulated osteoblast differentiation of human MSCs and stimulated osseointegration in vivo [29,31,32]. Our findings here suggest a reciprocal role with Wnt16, mediated by miR-145-5p, which is an upstream regulator of sema3A [14]. Exogenous Wnt16 had no effect on sema3A production, nor did the blocking of endogenous Wnt16. However, Wnt16 stimulated the production of miR-145-5p in the 3D cultures. miR-145-5p blocked the stimulatory effect of osteogenic media on the expression of an osteoblast phenotype in MSCs cultured on TCPS. Moreover, the transfection of MSCs with miR-145-5p-mimic stimulated the production of Wnt16 and inhibited production of sema3a on the porous constructs. Finally, the transfection of MSCs with miR-145-5p inhibitor reduced the production of Wnt16 and stimulated production of sema3A. Taken together, these results support the hypothesis that Wnt16 provides a regulatory control on osteogenesis, so that osteoprogenitor cells can continue to proliferate as they are stimulated to differentiate in a surface and porosity mediated manner.

These findings support our previous observations examining the use of rhWnt16b to promote the osseointegration of machined Ti implants in rat metaphyseal bone defects [9]. When MSCs were cultured on microtextured, hydrophilic Ti surfaces, Wnt16 production was upregulated; treatment with exogenous rhWnt16b increased proliferation but reduced osteoblast differentiation and production of local factors associated with osteogenesis. Based on the report that Wnt16 regulates bone morphology in mice [11,12], we assessed the effects of Wnt16 on peri-implant bone formation in a rat model of disuse osteopenia. Wnt16 injections in vivo did not significantly alter whole bone morphology, but were effective at increasing the cortical bone to implant contact. These findings suggest that Wnt16 serves as a factor balancing cell proliferation and osteoblast differentiation induced by Wnt11 and Wnt5a. This equilibrium facilitates the proliferation of osteoprogenitor cells necessary for net new bone formation while preventing excessive osteoblast differentiation, mitigating the risk of osteopetrosis.

Furthermore, the addition or suppression of Wnt16 did not exert an influence on sema3A production on porous surfaces. However, the introduction of Wnt16 increased the expression of miR-145-5p, an upstream regulator of sema3A, on porous substrates. This suggests that Wnt16 and sema3A may act independently but share an overlapping signaling pathway. miR-145-5p was found to regulate mesenchymal cell differentiation by osteogenic media. The experimental findings also showed the differential effects of miR-145-5p modulation on Wnt16 and sema3A production. The introduction of miR-145-5p mimic resulted in increased Wnt16 production on porous substrates, highlighting miR-145-5p’s potential role as an upstream regulator of Wnt16. Notably, this effect was specific to porous substrates, indicating the substrate-dependent nature of miR-145-5p’s influence. Conversely, miR-145-5p was capable of decreasing sema3A levels on porous substrates, where miR-145-5p expression was increased by Wnt16 treatment.

These results hint at a complex interplay between miR-145-5p, Wnt16, and sema3A in the context of 3D surface-mediated osteogenesis. The miR-145-5p mimic increased Wnt16 production on porous substrates, but it did not exert a similar effect on TCPS or solid substrates. This substrate-dependent modulation emphasizes the context-specific nature of miR-145-5p’s involvement in the regulatory network, and this is the first study to show the miR-145-5p effect on Wnt16 production and sema3A production in the 3D porous Ti6Al4V environment.

The intricate balance between these molecular entities appears to be finely tuned to regulate cell proliferation and osteoblast differentiation. Previous data showed that sema3A increased Wnt16 production on 2D smooth titanium surfaces to a similar level as observed on microrough and hydrophilic titanium surfaces [9], indicating the interaction of sema3A and Wnt16 in titanium surface-mediated osteogenesis. Elucidating the precise mechanism through which Wnt16 acts in conjunction with, or independently of, sema3A would benefit from genetic manipulation techniques. CRISPR-Cas9-mediated knockout or the over-expression of Wnt16, sema3A, and miR-145-5p could uncover their individual contributions and potential synergistic effects on osteogenesis, which can be a future direction of this research.

The interplay between the growth of MSCs on 3D porous titanium alloy substrates and osteoclast activity was demonstrated by increased OPG production on porous Ti6Al4V, and was confirmed by our data. Osteoclast activity was inhibited by conditioned media collected from MSC cultures grown on 3D porous constructs. Previous literature has suggested that Wnt16, a key regulatory factor implicated in osteogenic processes, can enhance OPG production during the early stages of osteoprogenitor cell differentiation [10,11]. Our experimental approach involving the addition or blocking of Wnt16 on porous surfaces demonstrated a clear modulation of OPG production. Considering the regulatory influence of Wnt16 on OPG and the well-established role of OPG as an inhibitor of osteoclasts [33,34], our findings raise the intriguing possibility that Wnt16 might play a role in the indirect modulation of osteoclasts by MSCs on porous substrates. Moreover, it remains an open question whether Wnt16 produced by osteoblasts on porous substrates could also have a direct effect on osteoclasts. Wnt16 produced by mouse and human osteoblasts has been shown to affect osteoclasts in other studies [11]. The findings presented here emphasize the dynamic and interconnected nature of the bone microenvironment, where 3D porous substrates not only influence osteoblast behavior but may also orchestrate a broader regulatory network impacting bone remodeling processes.

Despite the valuable insights gained from this study, several limitations should be acknowledged. While we extensively explored the impact of 3D porous titanium alloy substrates on osteogenesis, our focus primarily centered on MSC differentiation and osteoblast behavior. However, a more comprehensive investigation into the effects on osteoclast activity, immune responses, and blood vessel formation is warranted. In particular, the influence of 3D porous structures on VEGF production was noted in our study, indicating a potential role in angiogenesis. These aspects are crucial components of successful osseointegration and contribute significantly to the overall performance of orthopedic and dental implants. Additionally, the current study focused on the effects of exogenous and endogenous Wnt16 on osteogenesis. While this sheds light on Wnt16’s regulatory role, further studies are needed to elucidate the precise signaling pathways and molecular interactions involved in the observed effects. Unraveling these intricate mechanisms will contribute to a more nuanced understanding of 3D substrate-mediated osteogenesis and provide potential targets for therapeutic interventions for compromised patients, who are vulnerable for implant failures [32,35].

## 5. Conclusions

The additively manufactured 3D structure used in this study significantly influences MSCs, promoting their differentiation into osteoblasts without osteogenic media, and factors produced by the MSCs inhibited osteoclast activity, indicating its role in modulating bone remodeling. The effect of the 3D architecture, when compared to the 2D constructs with a comparable surface topography and chemistry, demonstrates the intricate regulatory mechanisms governing osteogenesis on 3D porous titanium alloy implants. Wnt16 emerges as a key regulator, balancing cell proliferation and osteoblast differentiation, and acting independently of Wnt3a, Wnt11, and Wnt5a. Wnt16 increased miR-145 expression without affecting sema3A and miR-145-5p increased Wnt16 production and inhibited sema3A production. The crosstalk between Wnt16 and sema3A, along with miR-145’s impact, adds complexity to the molecular orchestration of 3D surface-mediated osteogenesis. While unveiling foundational insights, this research points to future directions for the broader implications of 3D substrates in influencing diverse cellular processes, contributing to advancements in implantology and implant design for enhanced clinical outcomes.

## Figures and Tables

**Table 1:** Gene-specific primers.

Gene	Primer Sequence
h_GAPDH_F	GCTCTCCAGAACATCATCC
h_GAPDH_R	TGCTTCACCACCTTCTTG
h_U6 F1	CTCGCTTCGGCAGCACA
h_U6 R1	AACGCTTCACGAATTTGCGT
h_WNT16_F	TCTCCATCTCTCCTACAGCTCC
h_WNT16_R	ACATCCAGTTTCCTTGGGCT
h_145-5p F	AGCCGGTCCAGTTTTCCCAGGA
h_145-5p R	GTGCAGGGTCCGAGGT

## Data Availability

The raw data required to reproduce these findings are available upon reasonable request from the corresponding author. The processed data required to reproduce these findings are available upon reasonable request from the corresponding author.

## Supplementary Materials

The following supporting information can be downloaded at: https://www.mdpi.com/article/10.3390/cells14030211/s1. Supplementary Figure S1: the morphology of solid and porous Ti6Al4V constructs fabricated by additive manufacturing before surface processing and sterilization by gamma irradiation; Supplementary Figure S2: SEM of MSCs grown on solid and a porous construct; Supplementary Figure S3: effect of culture time on the response of human bone MSCs to solid and porous Ti6Al4V constructs fabricated by additive manufacturing; Supplementary Figure S4: the response of human bone MSCs from Donor 2.
