# Supplementary material for: Non-Canonical Wnt16 and microRNA-145 Mediate the Response of Human Bone Marrow Stromal Cells to Additively Manufactured Porous 3-Dimensional Biomimetic Titanium–Aluminum–Vanadium Constructs
Source: Cells. 2025 Feb 1;14(3):211. doi: 10.3390/cells14030211 (PMC11816670; doi:10.3390/cells14030211)
Supplement: Supplementary file 1 [file cells-14-00211-s001.zip › cells-3359694-supplementary.pdf]

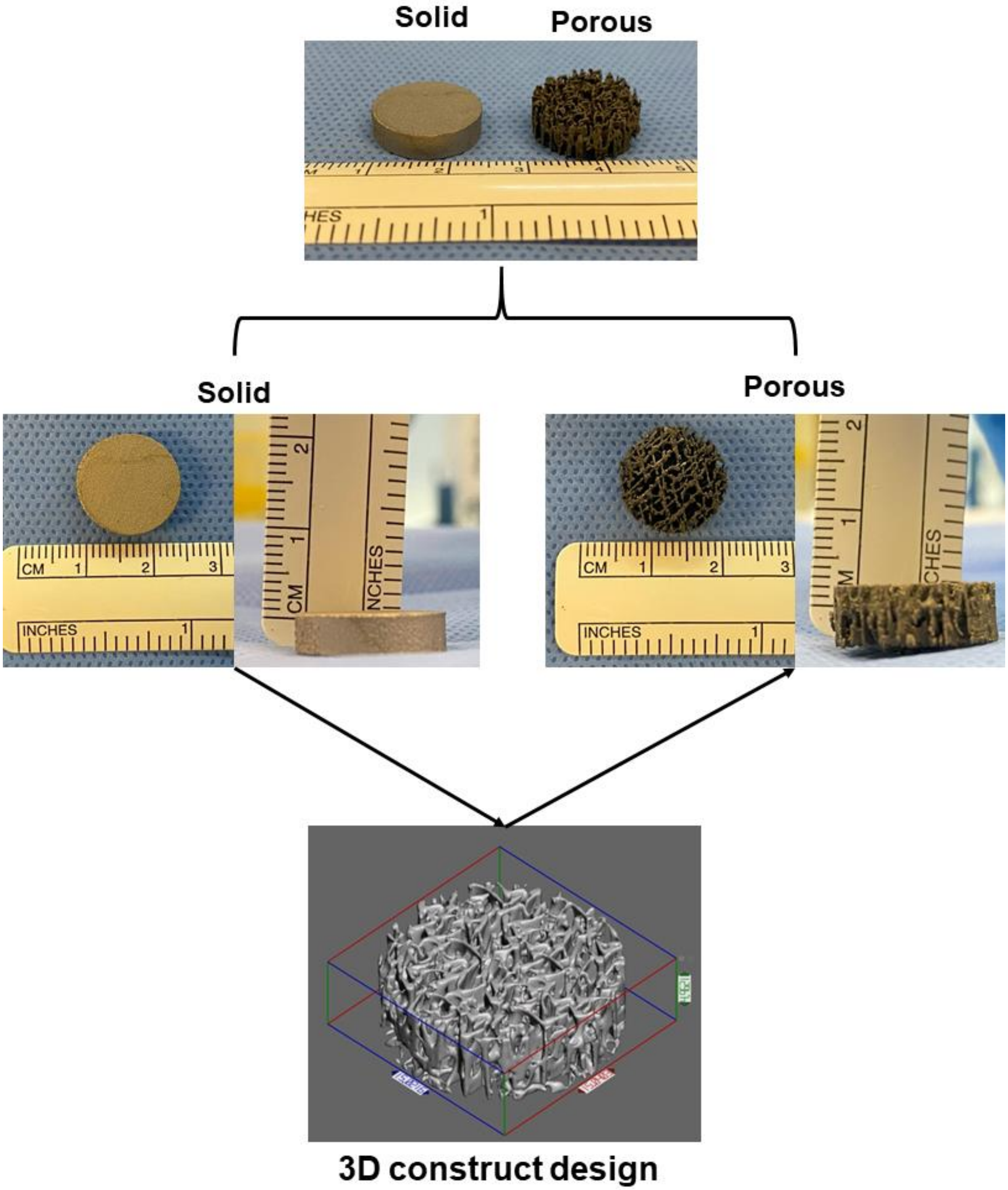

Supplemental Figure S1: The morphology of solid and porous Ti6Al4V constructs fabricated by additive manufacturing before surface processing and sterilization by gamma irradiation.

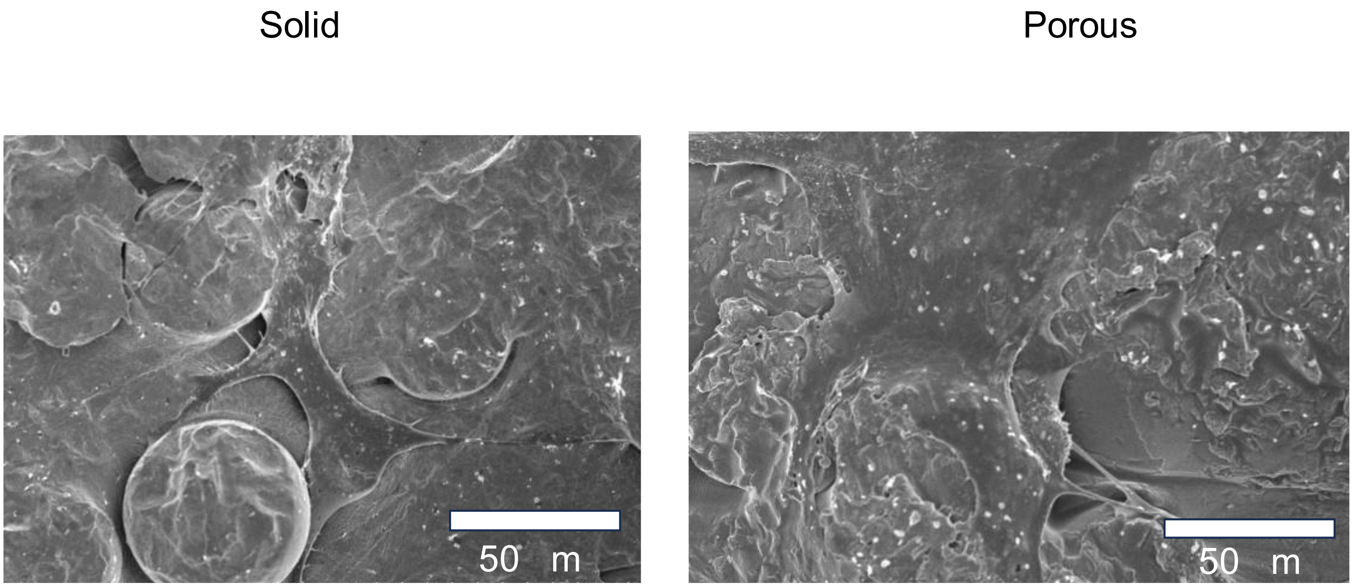

Supplemental Figure S2: SEM of MSCs cells grown on solid and porous constructs.

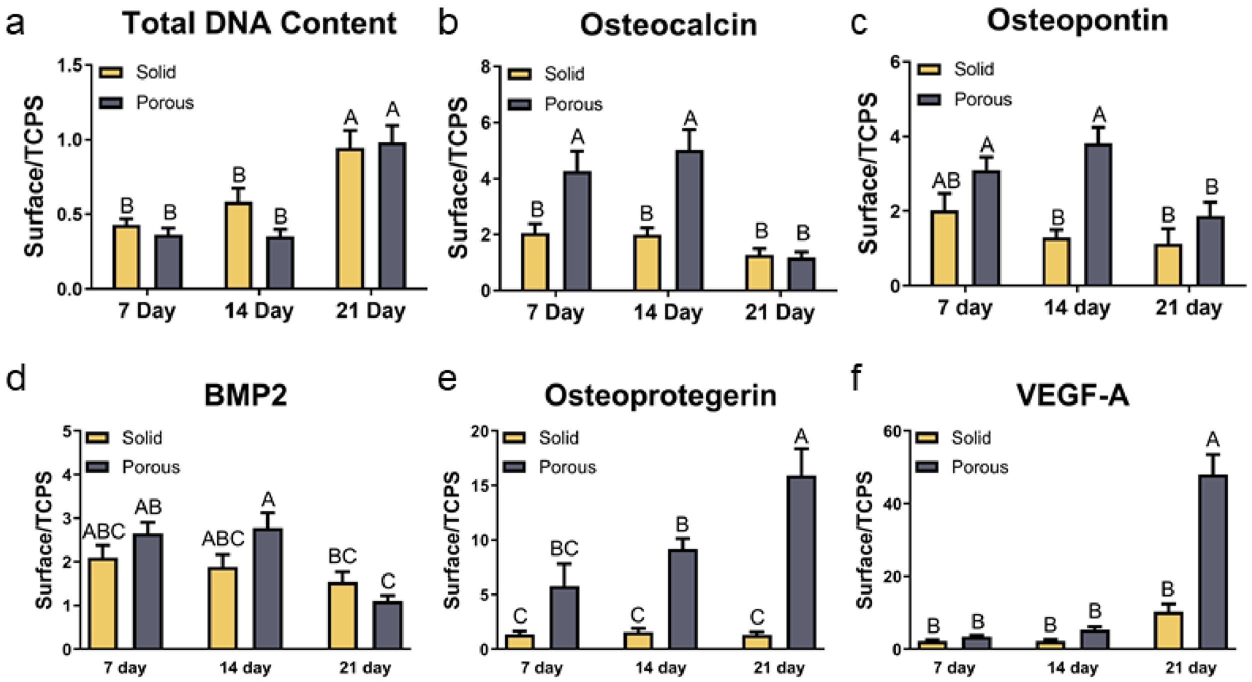

Supplemental Figure S3: Effect of culture time on the response of human bone MSCs to solid and porous Ti6Al4V constructs fabricated by additive manufacturing. MSCs were grown for 7, 14 and 21 days in growth media. (a) Total DNA content was determined in the cell layer lysate. (b) Osteocalcin, (c) osteopontin, (d) BMP2, (e) osteoprotegerin, and (f) vascular endothelial growth factor A were determined in the conditioned media after 24 hours. The data are presented as the ratio of values in the conditioned media of cells on the solid or porous surface compared to cultures grown on tissue culture polystyrene (TCPS). Data from one of at least two experiments are presented. Data are the means ± SEM, for N=6 independent cultures per variable. Groups not sharing the same letters are significant at $p<0.05$.

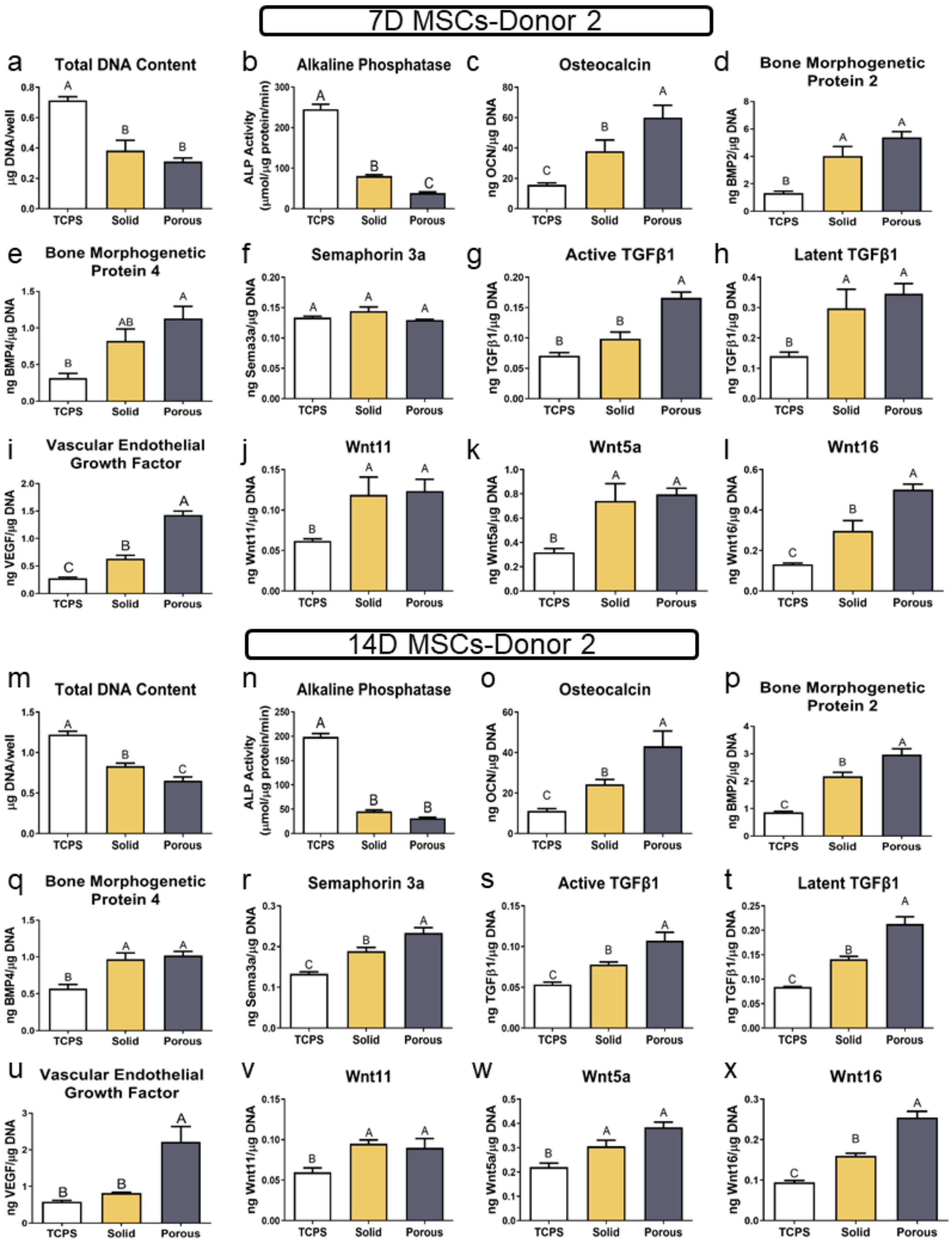

Supplemental Figure S4: The response of human bone MSCs from Donor 2 at 7 days (a-l) or 14 days (m-x) of culture on TCPS, solid Ti6Al4V constructs or porous Ti6Al4V constructs. (a, m) Total DNA content and (b, n) alkaline phosphatase specific activity was determined in the cell layer lysate. (c, o) Osteocalcin, (d, p) bone morphogenetic protein 2 (BMP2), (e, q) BMP 4, (f, r) semaphorin 3a, (g, s) active and (h, t) latent transforming growth factor beta 1, (i, u) vascular endothelial growth factor A, (j, v) Wnt11, (k, w) Wnt5a, and (l, x) Wnt16 were determined in the conditioned media after 24 hours. The data are normalized to DNA and are from one of at least two experiments. Data are the means ±SEM, for N=6 independent cultures per variable. Groups not sharing the same letters are significant at $p<0.05$.
